# Differential Expression of NADPH Oxidases Depends on Skeletal Muscle Fiber Type in Rats

**DOI:** 10.1155/2016/6738701

**Published:** 2016-10-26

**Authors:** Adriano César Carneiro Loureiro, Igor Coutinho do Rêgo-Monteiro, Ruy A. Louzada, Victor Hugo Ortenzi, Angélica Ponte de Aguiar, Ewerton Sousa de Abreu, João Paulo Albuquerque Cavalcanti-de-Albuquerque, Fabio Hecht, Ariclécio Cunha de Oliveira, Vânia Marilande Ceccatto, Rodrigo S. Fortunato, Denise P. Carvalho

**Affiliations:** ^1^Laboratório de Expressão Gênica, Instituto Superior de Ciências Biomédicas, Universidade Estadual do Ceará, Fortaleza, CE, Brazil; ^2^Laboratório de Radiobiologia Molecular, Instituto de Biofísica Carlos Chagas Filho, Universidade Federal do Rio de Janeiro, Rio de Janeiro, RJ, Brazil; ^3^Laboratório de Fisiologia Endócrina Doris Rosenthal, Instituto de Biofísica Carlos Chagas Filho, Universidade Federal do Rio de Janeiro, Rio de Janeiro, RJ, Brazil

## Abstract

NADPH oxidases (NOX) are important sources of reactive oxygen species (ROS) in skeletal muscle, being involved in excitation-contraction coupling. Thus, we aimed to investigate if NOX activity and expression in skeletal muscle are fiber type specific and the possible contribution of this difference to cellular oxidative stress. Oxygen consumption rate, NOX activity and mRNA levels, and the activity of catalase (CAT), glutathione peroxidase (GPX), and superoxide dismutase (SOD), as well as the reactive protein thiol levels, were measured in the soleus (SOL), red gastrocnemius (RG), and white gastrocnemius (WG) muscles of rats. RG showed higher oxygen consumption flow than SOL and WG, while SOL had higher oxygen consumption than WG. SOL showed higher NOX activity, as well as NOX2 and NOX4 mRNA levels, antioxidant enzymatic activities, and reactive protein thiol contents when compared to WG and RG. NOX activity and NOX4 mRNA levels as well as antioxidant enzymatic activities were higher in RG than in WG. Physical exercise increased NOX activity in SOL and RG, specifically NOX2 mRNA levels in RG and NOX4 mRNA levels in SOL. In conclusion, we demonstrated that NOX activity and expression differ according to the skeletal muscle fiber type, as well as antioxidant defense.

## 1. Introduction

Skeletal muscle fibers are constantly generating ROS in different subcellular locations, due to physiological and pathophysiological stimuli [1]. Previous studies have indicated mitochondria as the main source of ROS generation in skeletal muscle fibers, which is directly related to the consumption of oxygen by this organelle. Furthermore, it was recently demonstrated that ROS are generated in a controlled manner by enzymatic systems, such as NADPH oxidases (NOX) in muscle fibers, in response to physiological stimuli, which might play a role in muscle adaptations leading to the optimization of the contractile performance [2, 3].

The NOX family is composed of seven members, NOX1 to NOX5 and DUOX1 and DUOX2, which are differentially expressed among tissues. The main function of NOX enzymes is ROS production, and NOX1, NOX3, and NOX5 produce superoxide (O_2_^−^), while DUOX1 and DUOX2 and NOX4 seem to mainly generate hydrogen peroxide (H_2_O_2_) [4–6]. The physiological roles of NOX enzymes are quite diverse, as they act in a wide range of cellular processes, such as cellular proliferation, calcium release, and hormone biosynthesis, but their overexpression is associated with the pathophysiology of various diseases [7]. It has been shown that skeletal muscle fibers express both NOX2 and NOX4. NOX2 and its regulatory subunits and NOX4 are present in the sarcolemma, sarcoplasmic reticulum, and T tubules of muscle fibers. NOX4 was also found in the mitochondria, more specifically in the inner mitochondrial membranes [8]. NOX4 is constitutively active and does not require association with regulatory subunits, being mainly regulated by its expression levels [5, 8, 9]. NOX2 can be activated by specific agonists, such as angiotensin II, growth factors, and cytokines, as well as mechanical stress/contraction, which induce the association of regulatory subunits (p47phox, p67phox, p40phox, and Rac1) and the activation of this enzyme [9]. It has been reported that NOX enzymes play important physiological roles in skeletal muscle, being extremely relevant in muscle excitation-contraction coupling [9–12].

Muscle fibers can be grouped into three major categories: slow-twitch type I fibers with high capillary density and high oxidative capacity adapted to endurance exercise, fast-twitch fibers type IIb, which have low capillary density and low oxidative capacity ideal for sprint and anaerobic performance, and type IIa fibers that show intermediate characteristics [13]. NOX enzymes are implicated in the skeletal muscle excitation-contraction coupling; hence we hypothesized that a characteristic pattern of NOX expression might exist in the different skeletal muscle fiber subtypes. Thus, the aim of this study was to evaluate if NOX activity and expression in skeletal muscles are fiber type specific and the possible contribution of their differential expression to cellular oxidative stress and to the adaptation to aerobic training.

## 2. Material and Methods

### 2.1. Experimental Groups

Adult male Wistar rats weighing 250–300 g were maintained in an animal house with controlled lighting (12 h light-dark cycle) and temperature (23-24°C). This investigation conforms to the Guide for the Care and Use of Laboratory Animals published by the US National Institutes of Health (NIH Publication number 85-23, revised 1996) and was approved by the Institutional Committee for Evaluation of Animal Use in Research (Comissão de Ética com o Uso de Animais (CEUA) em Experimentação Científica da Universidade Estadual do Ceará, number 10724359-8-5).

### 2.2. Maximum Speed Testing

After acclimatization, animals underwent a test to determine the maximum speed race—Maximum Speed Test (MST). The test started at a speed of 0.3 km/h, and every 3 minutes 0.2 km/h of speed was added, increasing the velocity until animal exhaustion [14]. The animals that reached a speed between 1.7 and 2.7 km/h were selected to perform the exercise protocol.

### 2.3. Exercise Protocol

The training speed used was approximately 60% of Maximum Speed Testing (maximal lactate steady state). There were five training sessions per week. The animals trained 30 minutes daily during the first week, 1 hour daily in the second week, and 2 hours daily in the third week. After the experimental period, the animals were euthanized by decapitation. Rat tissues were dissected out, snap-frozen in liquid nitrogen, and then kept at −80°C until processing.

### 2.4. Oxygen Consumption in Permeabilized Fibers

Mitochondria respiration was studied* in situ* using saponin-permeabilized fibers, as previously described [15]. Muscles were removed and immediately immersed in cold BIOPS solution (10 mM EGTA, 0.1 *μ*M K-Mes, 0.5 mM DTT, 6.56 mM MgCl_2_, 5.77 mM ATP, and 15 mM phosphocreatine, pH 7.1). The fibers were separated and permeabilized for 30 min in iced BIOPS solution containing saponin (50 *μ*g/mL). After permeabilization, the fibers were washed for 10 min in a cold mitochondrial respiration MIR05 solution (0.5 mM EGTA, 3 mM MgCl_2_, 60 mM K-lactobionate, 20 mM taurine, 10 mM KH_2_PO_4_, 20 mM HEPES, 110 mM sucrose, and 1 g/L BSA, pH 7.1).

### 2.5. High-Resolution Respirometry

Skeletal muscle fibers (1.5–4.0 mg of muscle) were dried on filter paper, weighed, and placed on a high-resolution respirometer instrument chamber (Oxygraph-2k; Oroboros) with 2 mL of MIR05 solution at 37°C and left for 10 min for acclimatization. The substrate addition protocol to assess O_2_ flux was performed sequentially as follows: pyruvate (5 mM) and malate (5 mM), ADP (3 mM), cytochrome c (10 *μ*M), succinate (10 mM), oligomycin (1 *μ*g/mL), and KCN (10 mM) [15].

### 2.6. Citrate Synthase Activity

Muscle samples were homogenized in lysis buffer (50 mM sodium phosphate, 10% glycerol, 1% octal-phenol ethoxylate, 10 mM sodium orthovanadate, 10 mM sodium fluoride, and 10 mM sodium pyrophosphate, pH 7.4) and supplemented with protease inhibitor mixture (P8340; Sigma). After 30 min on ice, the tissue lysates were centrifuged at 13,000 ×g for 20 min at 4°C, and the resulting supernatants were collected. A reaction mixture containing 20 mM Tris·HCl, pH 8.0, 0.42 mM acetyl-coenzyme A, 0.1 mM 5′,5′-dithiobis(2-nitrobenzoic acid) (DTNB), and 5 *μ*g of total protein was incubated at 37°C for 5 min. The reaction was initiated by the addition of 0.5 mM oxaloacetate. The reduction of DTNB by citrate synthase was measured in a plate reader spectrofluorometer (Victor X4; PerkinElmer, Norwalk, CT) at 412 nm (extinction coefficient = 13.6 mM/cm corrected by the plate path length = 0.552 cm). The activities are expressed as micromoles of citrate per minute and milligram of protein [16].

### 2.7. NADPH Oxidase Activity

NOX activity was quantified in muscle microsomal fractions by the Amplex red/horseradish peroxidase assay, which detects the accumulation of a fluorescent oxidized product. In order to obtain the microsomal fraction, the homogenates from muscle samples were centrifuged at 3,000 ×g for 15 min at 4°C. Then, the supernatant was centrifuged at 100,000 ×g for 35 min at 4°C, and the pellet was suspended in 0.5 mL 50 mM sodium phosphate buffer, pH 7.2, containing 0.25 M sucrose, 2 mM MgCl_2_, 5 mg/mL aprotinin, and 34.8 mg/mL phenylmethanesulfonyl fluoride (PMSF) and stored at –80°C until H_2_O_2_ generation measurements. The microsomal fraction was incubated in 150 mM sodium phosphate buffer (pH 7.4), containing SOD (100 U/mL; Sigma, USA), horseradish peroxidase (0.5 U/mL; Roche, Indianapolis, IN), Amplex red (50 *μ*M; Molecular Probes, Eugene, OR), and 1 mM EGTA in the presence or absence of 1 mM NADPH. The fluorescence was immediately measured in a microplate reader (Victor X4; PerkinElmer, Norwalk, CT) at 30°C, using excitation at 530 nm and emission at 595 nm [17]. Specific NADPH oxidase activity was calculated by the differences between the activities in the presence and absence of NADPH.

The specific enzymatic activity was expressed as nmol H_2_O_2_ per hour and milligram of protein (nmol·h^−1^·mg^−1^). Protein concentration was determined by the Bradford assay [18].

### 2.8. Antioxidant Enzymes Activity

Muscle samples were homogenized in 5 mM Tris-HCl buffer (pH 7.4), containing 0.9% NaCl (w/v) and 1 mM EDTA, followed by centrifugation at 750 ×g for 10 minutes at 4°C. The supernatant aliquots were stored at −70°C. Catalase activity was assayed following the method of Aebi and was expressed as units per milligram of protein (U·mg^−1^) [19]. Glutathione peroxidase (GPX) activity was assayed by following NADPH oxidation at 340 nm in the presence of an excess of glutathione reductase, reduced glutathione, and tert-butyl hydroperoxide, as substrates [20], and expressed as nmol of oxidized NADPH per milligram of protein (nmol·mg^−1^). Total superoxide dismutase activity was determined by reduction of cytochrome C at 550 nm [21].

### 2.9. Measurement of Reactive Protein Thiols

Muscle samples were homogenized in 5 mM Tris-HCl buffer (pH 7.4), containing 0.9% NaCl (w/v) and 1 mM EDTA, followed by centrifugation at 750 ×g for 10 minutes at 4°C. The redox status of the studied tissues was determined by the measurement of reactive protein thiols immediately after sample homogenization. Total reduced thiols were determined using DTNB. Thiol residues react with DTNB, cleaving the disulfide bond to give 2-nitro-5-thiobenzoate (NTB^−^), which ionizes to the NTB^2−^ dianion in water at neutral and alkaline pH. NTB^2−^ was quantified in a spectrophotometer (Hitachi U-3300) by measuring the absorbance at 412 nm and was expressed as nmol of reduced DTNB/mg protein [22].

### 2.10. qPCR

Total RNA was extracted using RNeasy Mini kit (Qiagen, Venlo, Netherlands) and 2 *μ*g of RNA was used for reverse transcription with High Capacity cDNA Reverse Transcription Kit (Applied Biosystems, USA). Quantitative real-time PCR was performed with SYBR Green Master Mix (Thermo Scientific, Waltham, MA, USA). Data were analyzed using the 2^−ddCt^ method. Oligonucleotide sequences of NADPH oxidases and antioxidants enzymes are listed in Table 1. Oligonucleotide sequences used to characterize the types of muscle fibers are in Supplementary Table  1 (Table S1 in Supplementary Material available online at http://dx.doi.org/10.1155/2016/6738701). GUS was used as the control housekeeping gene.

### 2.11. Statistical Analysis

All results are expressed as mean ± standard error of the mean and were analyzed by One-Way ANOVA followed by Bonferroni's Multiple Comparison Test. Statistical analyses were done using GraphPad Prism (version 5.01, GraphPad Software Inc., San Diego, USA) and the minimum level of significance was set at *p* < 0.05.

## 3. Results

### 3.1. Characterization of Skeletal Muscle Fiber Types

First, we evaluated by qPCR the expression of hallmark genes related to muscle contraction, energy metabolism, and Ca^2+^ handling in order to better characterize the types of skeletal muscle fibers studied (Table 2). The slow isoform of myosin heavy chain (MHC1) was higher in soleus slow-twitch type I muscle (SOL), while type IIa red gastrocnemius (RG) had higher expression of the intermediate myosin heavy chain isoforms (MHC2a and MHC2x), and MHC2b isoform was the predominant one in fast-twitch type IIb white gastrocnemius (WG). In relation to genes involved in energy metabolism, PGC-1*α* and UCP3 that are important regulators of mitochondrial function were more expressed in RG and SOL, when compared with WG. However, genes involved in glucose metabolism, such as GDP1 and GDP2 expressions, were higher in WG. Finally, the expression of SERCA1 was greater in WG, while SERCA2 was more expressed in SOL.

**Figure 1:**
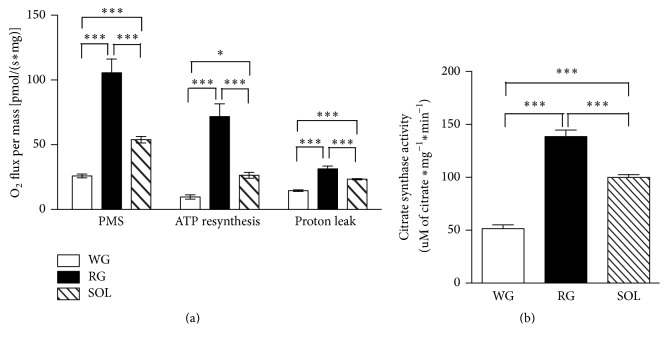
Basal levels of oxygen (O_2_) consumption and citrate synthase activity in slow- and fast-twitch oxidative and glycolytic skeletal muscle fibers of rats. (a) Maximal coupled O_2_ consumption after pyruvate, malate, and succinate (PMS) addition and proton leak and O_2_ consumption related to ATP synthesis and (b) citrate synthase activity. Data were obtained with 10 animals from at least two independent experiments and are shown as mean ± SEM. ^*∗*^ *p* < 0.05; ^*∗∗∗*^ *p* < 0.001.

Another parameter used in the characterization of skeletal muscle fiber types was their oxygen consumption capacity. We observed that WG consumed less oxygen than RG and SOL after stimulation with malate, pyruvate, and succinate (Figure 1(a)). In addition, the RG showed higher oxygen consumption rate related to ATP synthesis and the proton leak (Figure 1(a)). The known mitochondrial density marker citrate synthase activity was higher in RG followed by SOL and WG (Figure 1(b)).

Thus, based on our results we classified the muscles as follows: WG as fast-twitch glycolytic, RG as fast-twitch oxidative, and SOL as slow-twitch oxidative muscles.

### 3.2. NADPH Oxidase Activity and mRNA Basal Levels in Slow- and Fast-Twitch Oxidative and Glycolytic Muscles

**Figure 2:**
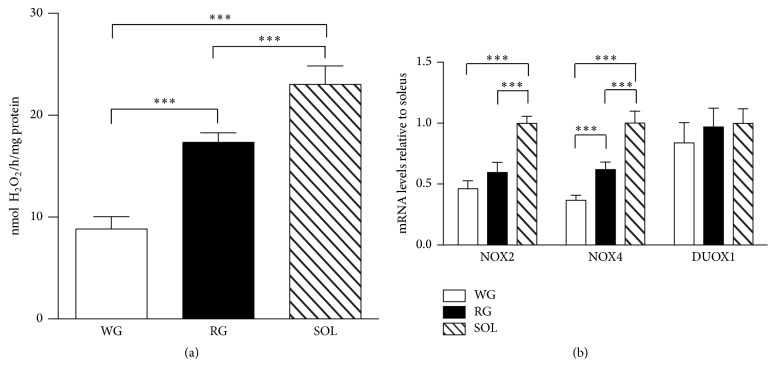
NADPH oxidase activity (a) and mRNA (b) basal levels of rat skeletal muscles. H_2_O_2_ production was determined in the microsomal fraction by the Amplex red/horseradish peroxidase assay. mRNA levels were determined by qPCR and were expressed relative to soleus muscle. Data were obtained with 10 animals from at least two independent experiments and are shown as mean ± SEM. ^*∗∗∗*^ *p* < 0.001.

NOX activity was significantly higher in SOL muscle in comparison to RG and WG; moreover, NOX activity was higher in RG when compared to WG (Figure 2(a)). In order to better characterize the NOX isoforms responsible for the higher H_2_O_2_ generation in oxidative muscle fibers, we evaluated the mRNA expression of the NOX enzymes. As previously stated, only NOX2, NOX4, and DUOX1 were detected in this tissue. NOX2 mRNA levels were higher in SOL muscle, but no differences were detected between WG and RG (Figure 2(b)). SOL fibers presented the highest NOX4 mRNA levels, when compared to RG and WG, while WG presented the lowest levels (Figure 2(b)). No differences in DUOX1 mRNA levels were observed among the muscle fibers analyzed (Figure 2(b)).

### 3.3. Basal Levels of Antioxidant Enzymes Activities in Slow- and Fast-Twitch Oxidative and Glycolytic Muscles

**Figure 3:**
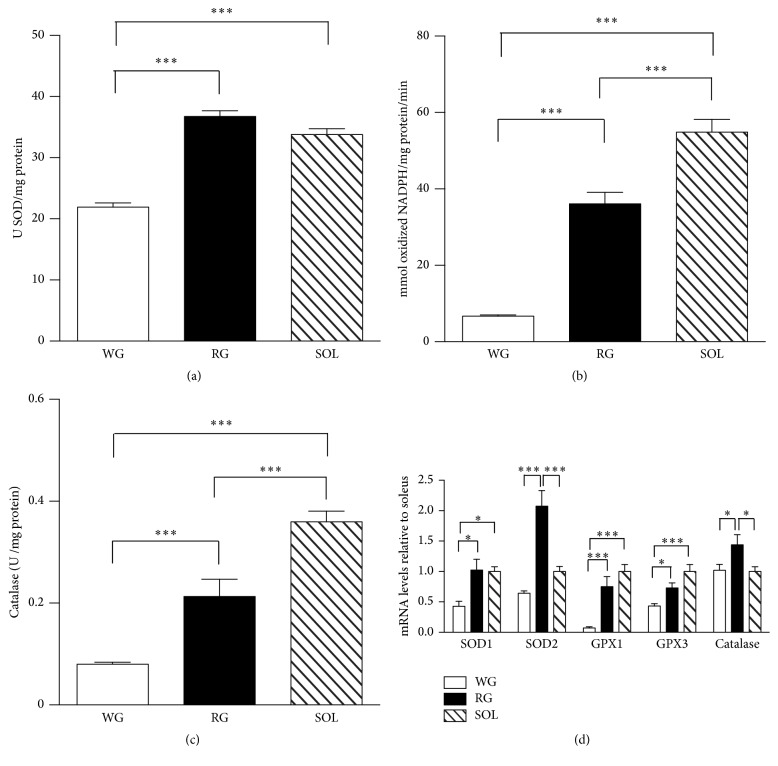
Basal levels of antioxidant enzymes activities and mRNA levels of rat skeletal muscles. Superoxide dismutase (a), glutathione peroxidase (b), and catalase (c) activities were measured by spectrophotometry. mRNA levels (d) were determined by qPCR and were expressed relative to soleus muscle. Data were obtained with 10 animals from at least two independent experiments and are shown as mean ± SEM. ^*∗*^ *p* < 0.05; ^*∗∗∗*^ *p* < 0.001.

Total SOD activity was significantly lower in WG in comparison to SOL muscle and RG (Figure 3(a)). In relation to GPX (Figure 3(b)) and catalase activities (Figure 3(c)), we observed a higher activity of both enzymes in SOL muscle fibers, when compared to RG and WG. Besides, GPX and catalase activities were higher in RG than in WG. The mRNA levels of the antioxidant enzymes, except catalase, are in accordance with the enzymatic activity levels (Figure 3(d)).

### 3.4. Basal Levels of Reactive Protein Thiols in Slow- and Fast-Twitch Oxidative and Glycolytic Fibers

**Figure 4:**
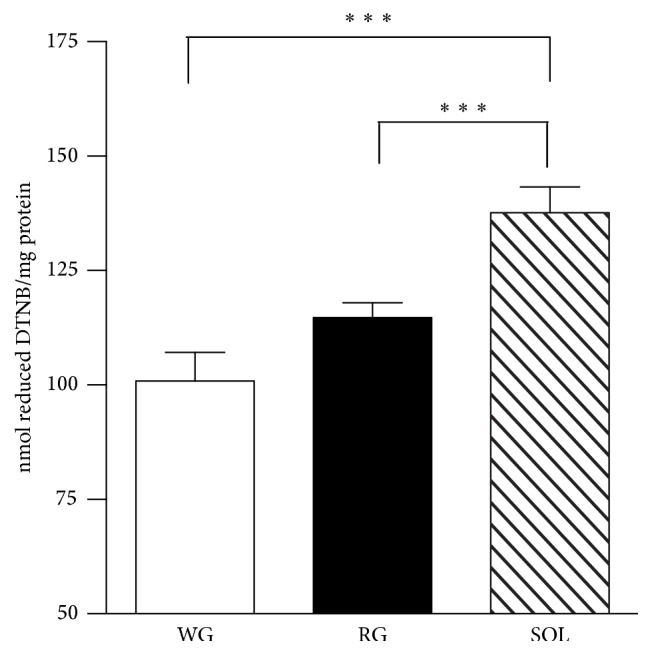
Basal levels of reactive protein thiol levels of rat skeletal muscles. Total sulfhydryl groups were measured by the reaction of thiols with DTNB, evaluated in a spectrophotometer at 412 nm. Data were obtained with 10 animals from at least two independent experiments and are shown as mean ± SEM. ^*∗∗∗*^ *p* < 0.001.

Thiol residues are mainly found in proteins and in low-molecular-mass metabolites, such as the highly abundant glutathione (GSH), and can be reversibly oxidized by ROS to nitrosothiols or sulfenic acids, decreasing their cellular levels. Interestingly, reactive protein thiol levels were higher in SOL in comparison to RG and WG and did not differ between RG and WG (Figure 4).

### 3.5. Effect of Physical Exercise on NOX Activity and mRNA Levels in Slow- and Fast-Twitch Oxidative and Glycolytic Muscles

**Figure 5:**
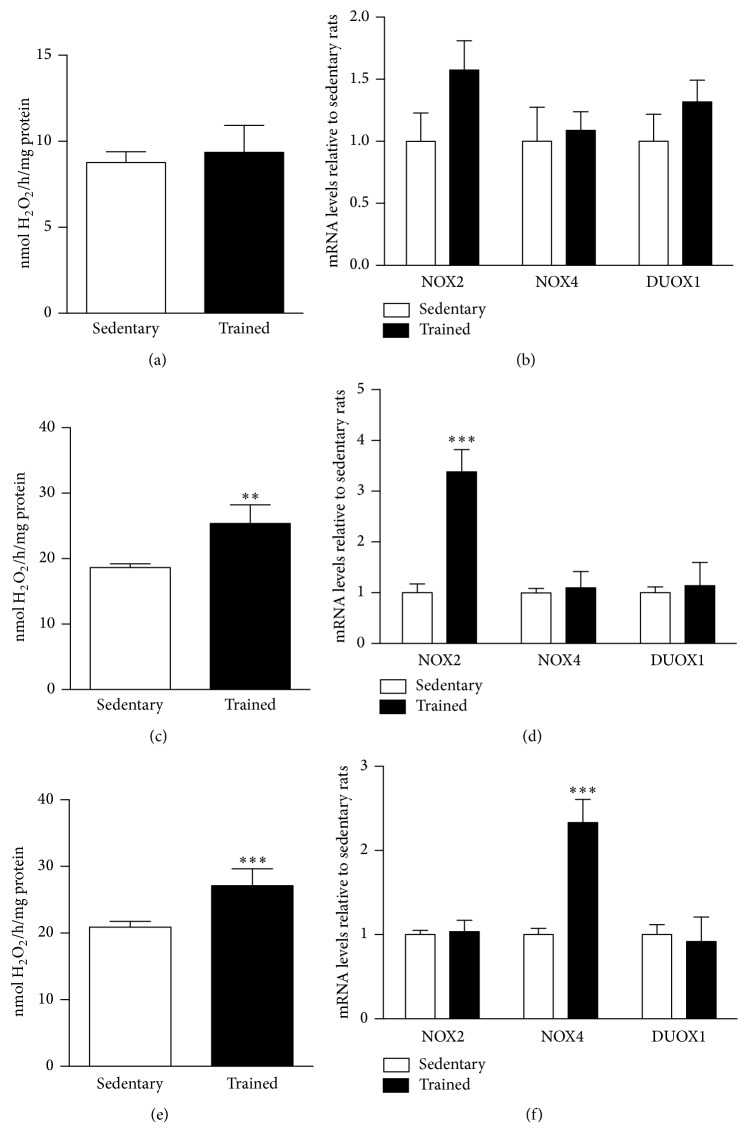
NADPH oxidase activity and mRNA levels of rat white gastrocnemius (a, b), red gastrocnemius (c, d), and soleus (e, f) skeletal muscles. H_2_O_2_ production was determined in the microsomal fraction by the Amplex red/horseradish peroxidase assay. mRNA levels were determined by qPCR and were expressed relative to soleus muscle. Data were obtained with 10 animals from at least two independent experiments and are shown as mean ± SEM. ^*∗∗*^ *p* < 0.01; ^*∗∗∗*^ *p* < 0.001.

After physical exercise training, NOX activity was significantly increased in SOL (Figure 5(e)) and RG (Figure 5(c)), but no differences were observed in WG (Figure 5(a)). As expected, there were no differences in the expression levels of NOX2, NOX4, and DUOX1 mRNA levels in WG (Figure 5(f)). However, NOX2 mRNA levels increased in RG of trained group, while NOX4 and DUOX1 mRNA levels were unchanged (Figure 5(d)). In SOL, NOX4 mRNA levels were higher in trained group in comparison to the control group, while NOX2 and DUOX1 mRNA levels remained unchanged (Figure 5(b)).

## 4. Discussion

To our knowledge, this is the first study that compares NOX expression and activity among different muscle fiber types. Our results raise important questions related to the redox homeostasis of skeletal muscle: (1) NOX expression and activity are higher in oxidative slow-twitch type I skeletal muscle fibers, (2) the increased NOX-derived ROS production found in oxidative slow-twitch type I skeletal muscle seems to be counterbalanced by a higher antioxidant defense, and (3) depending on the physiological stimulus, NOX enzymes can be modulated and might contribute to skeletal muscle adaptations through ROS generation.

Firstly, we showed that RG consumes more oxygen in all tested conditions than SOL and GB, while SOL consumes more oxygen than GB. Previous studies indicate that mitochondrial density is higher in oxidative slow-twitch type I fibers than in type II fibers [23, 24], being characterized by the high amounts of myoglobin and oxidative enzymes, which allow them to sustain longer contractile activities. White fast-twitch type IIb fibers have high metabolic activity with the predominance of the glycolytic pathway [23–25].

Residual oxygen consumption in isolated fibers, which is also called the nonmitochondrial oxygen consumption, although nonsignificant, tended to be higher in SOL. This result prompted us to evaluate the possible contribution of NOX enzymes to the different rate of oxygen consumption found in the different fiber types. It has been shown that skeletal muscles express NOX enzymes [5, 8, 9], but no previous studies compared the expression and activity of these enzymes among different skeletal muscle fiber types. NOX activity was significantly higher in SOL, followed by RG and WG. As NOX activity measurement does not discriminate the NOX isoform, we evaluated NOX expression in these tissues, and we found higher NOX2 and NOX4 expression levels in SOL in comparison to RG and WG. Moreover, NOX4 expression was higher in RG when compared to WG. Under physiological conditions, NOX2 and NOX4 seem to be involved in the skeletal muscle contraction mechanism. Sun et al. showed that NOX4 activity is positively modulated by muscle oxygen tension, increasing H_2_O_2_ availability, which oxidizes multiple cysteine residues of dihydropyridine receptor (RyR1) [10]. Then, this process promotes the release of Ca^2+^ from sarcoplasmic reticulum, which is crucial for muscle contraction. On the other hand, NOX2 seems to be activated by muscle contraction, when the mechanical stretching activates Rac1 protein, promoting NOX2 activation [9]. NOX2 derived ROS sensitizes transient receptor potential (TRP) channels that are located in the sarcolemma of skeletal muscle, increasing the influx of Ca^2+^, which is essential for maintaining SR loads during repetitive contractions [9, 11]. Another study shows that the RyR1 can be modified by S-glutathionylation through NOX2, located at T tubules membranes, promoting the release of Ca^2+^ from SR [12]. These data suggest an important physiological role of NOX enzymes in skeletal muscle excitation-contraction coupling. So, we can speculate that the differences regarding NOX expression found in these fiber types could be related to the contractile characteristics of each fiber. Thus, the higher NOX activity in SOL could be related to higher intracellular Ca^2+^ availability during contraction, which is crucial for sustaining muscle contraction for longer periods.

As discussed before, ROS production is important for skeletal muscle physiology, but increased ROS production can be deleterious due to the oxidation of macromolecules, such as lipids, nucleic acids, and proteins. So, cells have antioxidant defense mechanisms in order to protect them against the deleterious effects of ROS. As a result, higher ROS availability may result in the specific activation of redox-sensitive transcription factors, such as activator protein-1 (AP-1) and nuclear factor kappa B (NF-*κ*B). These factors regulate the expression of genes encoding several antioxidant enzymes, such as catalase, GPX, SOD1, and SOD2 [26–28]. Our results are in agreement with this hypothesis, since the antioxidant activity was directly correlated with NOX activity in these different muscle fiber types. The antioxidant defense seems to be crucial for maintaining muscle redox homeostasis, preventing cellular oxidative stress. We found that reactive protein thiol levels, a biomarker of the redox status, did not differ between RG and WG and were surprisingly higher in SOL muscle, which presented the highest NOX activity. These results demonstrate that in the muscle fibers that show higher ROS production the redox homeostasis is maintained, probably due to the differences in their antioxidant defense ability.

Skeletal muscle redox imbalance favors the accumulation of ROS and is associated with loss of muscle mass, inflammation, and degenerative diseases, such as sarcopenia and muscular dystrophies [29–31]. Sarcopenia is defined as the decline of muscle mass and/or function, generally promoted by aging [32, 33]. A study with female Balb/c mice of different ages showed that NOX2 and NOX4 were higher in the quadriceps of older animals, related to the onset of sarcopenia. Increased SOD activity and decreased catalase and GPX activity were also observed in older animals. These results suggest that the NOX enzymes could be related to the increased production of ROS in aged muscles. In addition, the higher ROS production, accompanied by the loss of antioxidant defense, could be considered important element to trigger the onset of sarcopenia [34].

Muscular dystrophies are inherited disorders and have been associated with the overexpression of NOX2, NOX4, DUOX1, and p47phox [35, 36]. Gene mutations encoding components of the dystroglycan complex, such as dystrophin and sarcoglycan, result in increased fragility of the sarcolemma [35]. Duchenne muscular dystrophy (DMD) is caused by dystrophin deficiency, and it was previously reported that DMD is related to high levels of oxidative damage in skeletal muscle, raising the question that ROS is implicated in its pathogenesis [37]. In mdx mice, a model for DMD, there is an increase in NOX4 and DUOX1 enzymes, with consequent increase in nitrotyrosine and 8-hydroxy-2′-deoxyguanosine. Interestingly, antioxidant administration decreased ROS levels and significantly reduced the oxidative damage and the muscle injury found in this pathology [35, 38]. Thus, although essential for skeletal muscle physiology, the excess of ROS can dramatically alter muscle function, being involved in a wide range of pathologies. Here we show that although ROS generated by NOX is higher in SOL, redox homeostasis is maintained by the antioxidant defense, reinforcing the physiological role of NOX in these tissues.

Several evidences show that ROS are able to change the processes of myogenesis* in vitro.* In this context, it has been shown that proliferation, differentiation, and fusion of myoblasts into myotubes can be adversely affected by excessive ROS [39]. Recently, it has been shown that myoblasts grown at 20% oxygen have higher ROS levels when compared to myoblasts grown at 6% [40]. As the different muscle types have important differences in vascularity, we cannot exclude the possibility that* in vivo* the activity of NOX could be even higher in muscles that are exposed to greater oxygen levels, as the SOL and RG, when compared to muscles less vascularized as the WG.

As NOX enzymes play important roles in muscle physiology, we speculated that their activities are modulated by physical exercise. In fact, in the present report we have shown that aerobic physical exercise training was able to increase NOX activity only in the SOL and RG fibers, with no differences in WG. It is important to note that the animals trained at 60% of maximum running speed testing, which corresponds to its maximal lactate steady state (MLSS) [41, 42]. MLSS is highly correlated with maximal aerobic power and performance of athletic endurance, being considered an excellent marker to determine the intensity of metabolic transition from aerobic and anaerobic pathways in continuous exercises [43, 44]. Thus, we can infer that the animals trained at their limit of aerobic metabolism intensity, which was sustained for a long period. Interestingly, only the WG did not adapt to this physical exercise protocol regarding NOX activity and expression, probably due to a decreased participation of this muscle fiber in this type of exercise.

The role of ROS in promoting muscle adaptive responses to training is well known. Indeed, PGC-1*α* has emerged as the mechanistic link to the best-known beneficial effects of exercise in muscle: mitochondrial biogenesis, angiogenesis, fatty acid utilization, and fiber type switching [45]. An important pathway triggered by ROS is the upregulation of the mitochondrial biogenesis master gene PGC-1*α*, but the source of ROS responsible for the increased PGC-1*α* after exercise was not demonstrated [46]. So, we hypothesize that an increased NOX activity in skeletal muscle could be related to the increased PGC-1*α* found after an acute session of exercise or physical training. Our results show that muscles that have higher baseline levels of PGC-1*α* also show higher levels and activity of NADPH oxidases (NOX2 and NOX4), suggesting that ROS generated by NADPH oxidases can contribute to the stability of PGC-1*α* mRNA and may participate in the maintenance of higher PGC-1*α* levels found in SOL and RG muscles, when compared to the WG [47].

In conclusion, we demonstrated that NOX activity and expression differ depending on the skeletal muscle fiber type, being higher in the fibers that carry a more sustained contraction. Despite the differences in ROS generation, muscle redox homeostasis is probably maintained due to the increased antioxidant activity found in muscles that show higher ROS generation ability. Taken together, our findings point out the potential role of NOX enzymes in skeletal muscle physiology and in their adaptation to exercise training.

## Supplementary Material

Oligonucleotide sequences used to characterize the types of skeletal muscle fibers.

## Figures and Tables

**Table 1:** Primers sequence using in RT PCR.

Gene	Forward	Reverse
Catalase	5′ CAAGCTGGTTAATGCGAATGG 3′	5′ TTGAAAAGATCTCGGAGGCC 3′
GPX1	5′ AATCAGTTCGGACATCAGGAG 3′	5′ GAAGGTAAAGAGCGGGTGAG 3′
GPX2	5′ TCCCTTGCAACCAGTTCG 3′	5′ TCTGCCCATTGACATCACAC 3′
GPX3	5′ CAGCTACTGAGGTCTGACAG 3′	5′ ACTAGGCAGGATCTCCGAG 3′
SOD1	5′ TGTGTCCATTGAAGATCGTGTG 3′	5′ CTTCCAGCATTTCCAGTCTTTG 3′
SOD2	5′ GGACAAACCTGAGCCCTAAG 3′	5′ CAAAAGACCCAAAGTCACGC 3′
SOD3	5′ GACCTGGAGATCTGGATGGA 3′	5′ GTGGTTGGAGGTGTTGTGCT 3′
NOX2	5′ CAATTCACACCATTGCACATC 3′	5′ CGAGTCACAGCCACATACAG 3′
NOX4	5′ TCCATCAAGCCAAGATTCTGAG 3′	5′ GGTTTCCAGTCATCCAGTAGAG 3′
DUOX1	5′ GATACCCAAAGCTGTACCTCG 3′	5′ GTCCTTGTCACCCAGATGAAG 3′
GUS	5′ GGTCGTGATGTGGTCCTGTC 3′	5′ TGTCTGCGTCATATCTGGTATTG 3′

GPX: glutathione peroxidase; SOD: superoxide dismutase; NOX: NADPH oxidase.

DUOX1: dual oxidase 1; GUS: beta glucuronidase.

**Table 2:** Real-time qPCR. Values were calculated to compare mRNA expression pattern level of genes in rat skeletal muscle. Values were normalized for soleus.

	WG	RG	SOL
*MHC*			
MHC1	0.0004	0.12	**1**
MHC2a	0.03	**58.8**	1
MHC2x	149	**788.2**	1
MHC2b	**11405**	320	1
*Metabolism*			
PGC1*α*	0.58	**2.1**	1
UCP3	0.54	**3.4**	1
GDP1	**20.4**	9.3	1
GDP2	**10.3**	8.1	1
*Ca* ^*2+ *^ *metabolism*			
SERCA1	**18.4**	10.3	1
SERCA2	0.0041	0.3	**1**

Data shown as mean of 5–8 rats.
