# Supplementary figures and images for: Differential Expression of NADPH Oxidases Depends on Skeletal Muscle Fiber Type in Rats
Source: Oxid Med Cell Longev. 2016 Oct 26;2016:6738701. doi: 10.1155/2016/6738701 (PMC5101397; doi:10.1155/2016/6738701)

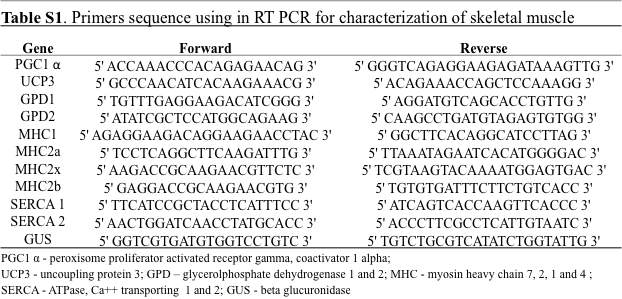

Supplement: Supplementary file 1 — Oligonucleotide sequences used to characterize the types of skeletal muscle fibers. [file 6738701.f1.doc]
